# Structural Gray Matter Changes in the Hippocampus and the Primary Motor Cortex on An-Hour-to-One- Day Scale Can Predict Arm-Reaching Performance Improvement

**DOI:** 10.3389/fnhum.2018.00209

**Published:** 2018-06-08

**Authors:** Midori Kodama, Takashi Ono, Fumio Yamashita, Hiroki Ebata, Meigen Liu, Shoko Kasuga, Junichi Ushiba

**Affiliations:** ^1^Graduate School of Science and Technology, Keio University, Yokohama, Japan; ^2^Saiseikai Higashikanagawa Rehabilitation Hospital, Yokohama, Japan; ^3^Institute for Biomedical Sciences, Iwate Medical University, Iwate, Japan; ^4^Department of Rehabilitation Medicine, Keio University School of Medicine, Tokyo, Japan; ^5^Centre for Neuroscience Studies, Queen’s University, Kingston, ON, Canada; ^6^Keio Institute of Pure and Applied Sciences, Yokohama, Japan; ^7^Department of Biosciences and Informatics, Faculty of Science and Technology, Keio University, Yokohama, Japan

**Keywords:** arm-reaching, longitudinal study, mirror-reversal transformation, rapid plasticity, voxel-based morphometry

## Abstract

Recent studies have revealed rapid (e.g., hours to days) training-induced cortical structural changes using magnetic resonance imaging (MRI). Currently, there is great interest in studying how such a rapid brain structural change affects behavioral improvement. Structural reorganization contributes to memory or enhanced information processing in the brain and may increase its capability of skill learning. If the gray matter (GM) is capable of such rapid structural reorganization upon training, the extent of volume increase may characterize the learning process. To shed light on this issue, we conducted a case series study of 5-day visuomotor learning using neuroanatomical imaging, and analyzed the effect of rapid brain structural change on motor performance improvement via regression analysis. Participants performed an upper-arm reaching task under left-right mirror-reversal for five consecutive days; T1-weighted MR imaging was performed before training, after the first and fifth days, and 1 week and 1 month after training. We detected increase in GM volume on the first day (i.e., a few hours after the first training session) in the primary motor cortex (M1), primary sensory cortex (S1), and in the hippocampal areas. Notably, regression analysis revealed that individual differences in such short-term increases were associated with the learning levels after 5 days of training. These results suggest that GM structural changes are not simply a footprint of previous motor learning but have some relationship with future motor learning. In conclusion, the present study provides new insight into the role of structural changes in causing functional changes during motor learning.

## Introduction

The adult brain has a remarkable ability for learning new motor skills and adapting to novel environments (Dayan and Cohen, 2011). In addition to behavioral or computational approaches to reveal the mechanism of a motor learning system (Shadmehr and Mussa-Ivaldi, 1994; Krakauer et al., 2000), much attention nowadays has been focused on the neuronal changes underlying behavioral changes. Especially, the development of magnetic resonance imaging (MRI) has enabled us to non-invasively investigate the functional and structural neurophysiological alterations resulting from motor learning. For example, Draganski et al. (2004) demonstrated an increase in the gray matter (GM) volume in the mid-temporal area and left posterior intraparietal sulcus after 3 months of juggling training. Subsequent investigations also showed potential structural changes in the human brain associated with motor training (Draganski et al., 2004; Boyke et al., 2008; Driemeyer et al., 2008; Taubert et al., 2010; Landi et al., 2011; Gryga et al., 2012; Sampaio-Baptista et al., 2014). In addition, a pioneering study reported the association between navigational experience and volume of the posterior hippocampus in taxi drivers, inferring structural plasticity induced by spatial learning (Maguire et al., 2000, 2006) Animal studies also have demonstrated that structural neural substrates contribute to motor skill learning and spatial navigation and memory (Yang et al., 2009, 2014; Sagi et al., 2012).

Recently, short-term brain plasticity in the period of hours to days was revealed, both in the white matter (Hofstetter et al., 2013) and GM (Sagi et al., 2012; Taubert et al., 2016). Such short-term brain plasticity is considered to represent cellular processes during motor learning subsequently contributing to synaptogenesis, such as astrocytic or microglial remodeling (Blumenfeld-Katzir et al., 2011; Lerch et al., 2011; Kassem et al., 2012; Sagi et al., 2012). Further, Taubert et al. (2016) demonstrated cortical thickness changes in such timescale occurred in a task-relevant manner (i.e., linearly increased across the training session). In addition, a previous study (Bailey and Chen, 1989), using the associative learning of *Aplysia californica*, demonstrated an increase in the number of varicosities and active zones 24–48 h after the training of sensitization, and the following time course of the structural changes was similar to the duration of the memory retention. Therefore, we hypothesized that short-term structural plasticity can occur in accordance with neural changes due to ongoing motor learning.

Further, the relationships between the degree of structural changes and behavioral performance improvement have been recently demonstrated. The GM changes before and after training correlated with the performance improvement over 5 days in a sequential pinch force task (Gryga et al., 2012), and with the final performance after 4 weeks of juggling training (Sampaio-Baptista et al., 2014). Based on the aforementioned studies, we hypothesized that short-term brain structural changes could predict an individual’s performance gains. Therefore, the purpose of the current study was to investigate how short-term structural changes induced by motor training contribute to further performance improvement in human participants.

To accomplish this, we adopted the model-based approach that has been used for computational motor learning research (Krakauer et al., 1999; Paz et al., 2003; Diedrichsen et al., 2005; Wolpert et al., 2011), because using this approach we can quantify the process of motor learning but not the performance at a specific time-point. We obtained behavioral data while participants performed visually guided arm-reaching training for 5 days. This task has an advantage over the model-based quantitative analysis for the motor learning process (Krakauer et al., 1999; Paz et al., 2003; Diedrichsen et al., 2005; Wolpert et al., 2011), compared with conventional tasks used for previous studies investigating the relationship between motor training and brain structural changes (Draganski et al., 2004; Taubert et al., 2010). To investigate short-term brain plasticity, MR scans were obtained after arm-reaching training on the first day of five continuous days of training, and whole brain analysis was performed using voxel-based morphometry (VBM). After determining the regions of interest where a significant volume increase was detected, we assessed how rapid GM structural changes in these regions affected further motor learning. To evaluate the association between GM structural changes and performance improvement, we performed multivariate regression analyses using model-based quantitative measures of task performance and the VBM results. We showed for the first time that the volume of GM increase in the regions related to visuomotor learning on the first day predicted the performance improvement after completing the entire training, providing new insight into the relationship between structural and functional changes during motor learning.

## Materials and Methods

### Study Design

The purpose of this pilot study was to investigate how and to what extent short-term structural changes induced by motor training contribute to further performance improvement. We therefore selected a regression experimental design (without control group) to extract structural variables that explain following motor performance.

### Participants

Fifteen healthy, right-handed participants (21.4 ± 1.4 years, 8 female subjects) participated in the study. The handedness of each participant was tested using the Edinburgh Inventory (Oldfield, 1971). *A priori* statistical test with Cohen’s *d* was performed to determine an appropriate sample size. The effect size was assumed to be 0.84, based on a previous reports (Gryga et al., 2012) which revealed the association between GM increase in the primary motor cortex (M1) and behavioral indexes. Since stepwise regression analysis eliminates the number of coefficients, we assumed the maximum and minimum number of coefficients. The sample size was selected such that the effect size for the multiple regression would have a power > 1 − *β* = 0.8, with α set at 0.05 (Bonferroni-corrected for five regression models). According to G*Power (Faul et al., 2007), the required minimum sample size was 8 for the minimum number of coefficients (= 1) and 17 for the maximum of coefficients (= 6). We recruited participants based on this range of sample size. Finally, 15 participants completed all the experiments without dropping out.

The participants reported normal or corrected-to-normal eyesight and did not have any neurological or psychiatric disorders. In addition, all participants were not smokers. This study was conducted in accordance with the Declaration of Helsinki, and the experimental procedures were approved by the ethical committees of Faculty of Science and Technology, Keio University. Written informed consent was obtained from all participants prior to experimentation. All participants participated in all the behavioral experiments and MR scanning sessions.

### Apparatus

**Figure 1:**
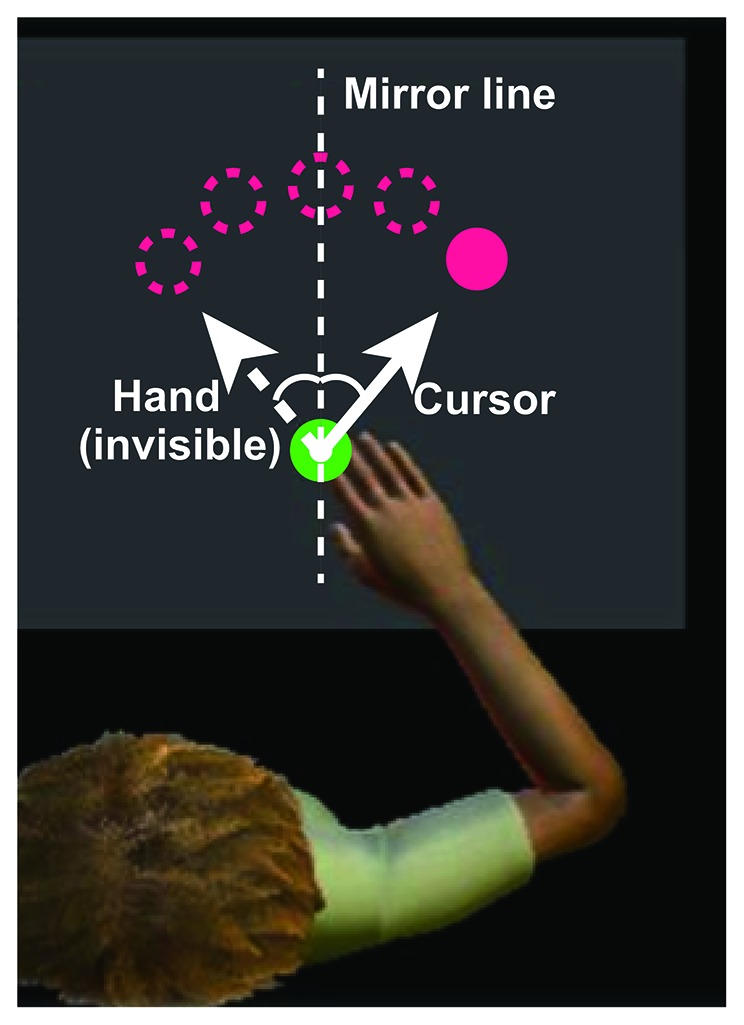
Experimental setup. During the experiments, visual information is displayed on a horizontal virtual reality display above the hand. The upper magenta circles indicate the targets, the green circle at the bottom of the display indicates the starting position, and the small white circle indicates the cursor. The solid and dotted lines indicate the cursor and hand paths, respectively. The x-coordinate of the cursor is obtained by flipping the sign of the x-coordinate of the fingertip, defining the starting position as the origin. Image modified from Dexterit-E Explorer 1.1 (BKIN Technologies Ltd., Kingston, ON, Canada).

Participants were instructed to perform a visually guided reaching task to a target using a robotic arm device (KINARM Exoskeleton, BKIN Technologies, Kingston, ON, Canada), permitting elbow and shoulder movement in the horizontal plane (Scott, 1999). Projected target lights and hand feedback were presented in the plane of the arm using a television monitor and semitransparent mirror. Participants were instructed to use their right hand to move a white circular cursor indicating the fingertip position (6 mm diameter) from the starting position (16 mm diameter) to the target (16 mm diameter), randomly presented at one of the five locations described below, on the virtual reality display (72 × 35 cm). The distance between the starting point and each target was 10 cm. The target was presented at 0°, ± 15°, and ± 30° positions. During the training task, the left-right relationship between the arm movement and visual feedback was reversed (i.e., mirror-reversal transformation; Figure 1). The position of the cursor was initially converted using an analog-digital converter at 1.129 kHz, and then re-sampled and recorded at 1 kHz for offline analysis.

### Visuomotor Task

The participants performed two sets of 150 trials under the mirror-reversal transformation for an hour on five consecutive days. Before the training task, the participants practiced approximately 150 trials without mirror-reversal transformation. Skill retention was assessed at 1 week and 1 month after the training period using the same procedure employed during the training task. Before each trial, they had to place the cursor at the starting point, and the gray target appeared 2 s later. After an additional randomly selected holding time (1–2 s), the target color changed to red (the “go” cue). In all tasks, the participants were instructed to perform ballistic reaching within 2 s of the target’s appearance. They were also required to maintain the peak velocities as constantly as possible across the trials. To facilitate this, a warning message was presented on the screen if the speed of the handle was either above (fast) or below (slow) the speed of 470 ± 45 mm/s. The speed range was defined based on the minimum jerk theory (Flash and Hogan, 1985), assuming that the participants move 10 cm for 400 ms.

### Behavioral Data Analysis

All trials where movement onset was detected after the go cue and offset was detected within 850 ms of onset were included in the subsequent offline analyses (850 ms indicates that the movement was too slow for 10 cm-reaching; Klassen et al., 2005). The position and velocity data of the hand obtained from KINARM was low-pass filtered using a zero-lag fourth-order Butterworth filter (cut off frequency, 8 Hz). Movement onset was defined as the time at which the hand velocity first exceeded 5% of the estimated peak velocity. Movement offset was defined as the time at which the hand velocity dropped below 5% of the estimated peak velocity for at least 100 ms. An initial angular error was defined as the absolute angle from a line connecting the starting position and the target to a line connecting the starting position and the point the cursor reached 150 ms after movement onset. An endpoint angular error was defined as the absolute angle from a line connecting the starting position and the target to a line connecting the starting position and the point where movement offset was detected. Counterclockwise and clockwise directions were defined using positive and negative directional values, respectively. For each participant, the errors of 300 trials for each day were averaged during offline analyses. To support the hypothesis that brain changes accompanying motor training are specifically associated with learning, we also calculated an averaged peak velocity across the 300 trials for each day as a control behavioral parameter that is independent of motor learning.

Behavioral data for each day were normally distributed (Kolmogorov-Smirnov test). A two-way repeated measures analysis of variance (ANOVA), with time and error types as factors, was performed to detect significant differences between the initial angular error and the endpoint angular error. Tukey’s honest significant difference (HSD) tests were performed to compare the first and last days of training. A paired *t*-test was performed to test the difference in the average peak velocity between the first and last days of training. The significance threshold was set at *P* < 0.05.

To further investigate the process of motor learning (i.e., learning speed and final error level), two kinds of errors of the participants during the training period (i.e., 1–5 days) were fitted with a simple exponential function (Equation 1):

(1) $$f(d)=Ae^{-\lambda d}+C$$

where *f* (*d*) is a learning curve as a function of day *d*; *A*, λ and *C* (constrained by *A* = 28.65 for initial error and 24.45 for endpoint error; λ > 0; *C* > 0) are the parameters determining error reduction, learning speed and final error level, respectively. First, we determined a fixed parameter *A* by fitting the data of an across-participants average of the initial or endpoint error calculated in a day-by-day manner using the exponential function (Equation 1). Then, for each participant, we fitted the data of the initial error, endpoint error, or peak velocity using the exponential function to estimate parameters λ and *C* (Table 1). We set the parameter *A* as a common constant across participants, because if we set all the three constants (*A*, λ and *C*) as free parameters, the results often converged to abnormal local minima or failed to converge. Compared with λ and *C*, the variability of parameter *A* across participants can be relatively constrained due to the experimental setup (i.e., mirror-reversal and the target positions). Therefore, we considered it reasonable to set *A* as a constant to avoid the aforementioned failure of model-fitting. In addition, using learning curves averaged across-participants to estimate the constant *A* would also average the bias between the participants.

**Table 1:** Parameters estimated using exponential fit.

Participant	Initial error				Endpoint error			
	*A*	*λ*	*C*	*R*^2^	*A*	*λ*	*C*	*R*^2^
All	28.65	0.8177	10.78	0.9994	24.45	0.9162	4.471	0.9924
A	28.65	0.9184	5.054	0.9905	24.45	3.281	4.822	0.0398
B	28.65	0.5479	11.92	0.9442	24.45	1.753	5.665	0.9021
C	28.65	0.9895	8.818	0.9144	24.45	0.6345	3.399	0.8323
D	28.65	0.9356	7.290	0.9470	24.45	0.6934	2.787	0.8005
E	28.65	0.6268	8.931	0.9583	24.45	1.072	5.169	0.9622
F	28.65	0.9798	9.995	0.9437	24.45	0.6222	4.114	0.9036
G	28.65	0.4406	6.442	0.9760	24.45	0.7053	2.618	0.8574
H	28.65	1.341	17.63	0.5276	24.45	0.7995	4.064	0.9485
I	28.65	1.162	9.704	0.8926	24.45	1.777	4.039	0.9722
J	28.65	0.1693	5.019	0.6294	24.45	1.194	4.034	0.2585
K	28.65	1.407	7.810	0.9216	24.45	2.119	3.392	0.9531
L	28.65	1.574	7.540	0.9333	24.45	1.904	4.501	0.9350
M	28.65	0.1407	7.494	0.8756	24.45	0.2337	3.178	0.7318
N	28.65	0.8371	9.299	0.9649	24.45	2.163	4.068	0.8466
O	28.65	0.4265	9.863	0.8394	24.45	0.4425	5.991	0.7313

*The top row of the parameters indicates the results estimated by the average data. We used *A* derived by fitting the curve, with the average data as a fixed parameter for the subsequent fitting for each individual participant*.

We used the exponential function, instead of other models (e.g., state-space model), to explain the behavioral changes because: (i) the computational learning model of mirror-reversal transformation is still under debate (Lillicrap et al., 2013); and (ii) the exponential function was sufficient to explain a gradual reduction of errors without *a priori* assumption regarding learning rules. A Mann-Whitney *U-test* was performed to detect a significant difference in the behavioral parameters (λ and *C*) between the male and female participants.

### MRI Data Acquisition

Data was acquired using a 1.5 T SIGNA EXCITE II scanner (GE Healthcare, Chalfont St. Giles, United Kingdom) using an 8-channel head coil. We used the same scanner, without any software updates, throughout the study period. In each scanning session, we acquired three axial T1-weighted anatomical images using a 3-dimensional fast spoiled gradient sequence (TR = 7.644 ms, TE = 3.144 ms, flip angle = 15°, voxel size = 1 × 1 × 1.4 mm^3^, field of view = 256 × 256 mm, 126 slices covered cerebellum). For each scanning session, the acquisition time of three T1 images was about 20 min. The MRI scans were obtained 4.7 ± 3.1 days (3–12 days) prior to the first day of training for baseline evaluation (i.e., prior to all behavioral measurements, including practice session without mirror-reversal transformation), 6.8 ± 1.8 h (3–8 h) after the daily training, on the first and fifth days, and 1 week and 1 month after the 5-day training protocol. The MRI scans on 1 week and 1 month after the last day of training were obtained prior to the behavioral retention test; thus, the retention tests did not affect the scans. All scans were performed in the evening. Three sets of images were lost due to data-saving issues (participant M on day 1, participants K and L on day 5). For statistical analyses, these cases were treated as missing values.

### MRI Data Processing and Analysis

First, three images for each participant, scanned on the same day, were co-registered and averaged to yield a single high-quality image for each time-point. For quality assessment, all images were visually checked, and those containing artifacts due to body movement were excluded during averaging of images. We excluded a volume image if the noise exceeded 10% of the area. The number of excluded volume images was five.

Pre-processing of T1-weighted images was performed using SPM8 (Wellcome Trust Center for Neuroimaging) and VBM8 Toolbox^1^ running in a MATLAB environment (MathWorks, version 8.0, Natick, MA, USA). The T1-weighted images were then processed using the “Process Longitudinal Data” pipeline implemented in the VBM8 Toolbox. In this pipeline, all longitudinal high-quality images for each participant were realigned, signal inhomogeneity-corrected, averaged, segmented for gray/white matter, and spatially normalized into a standard anatomical space. Spatial normalization was performed using DARTEL (Ashburner, 2007), applying a single deformation field estimated from a longitudinally averaged image to images for all time-point to preserve warping consistency. The warped GM images were scaled using the Jacobian determinants of the deformations to account for local compression and expansion during linear and nonlinear transformations. Finally, the modulated GM volumes were smoothed using a Gaussian kernel of 8 mm full-width at half maximum.

To detect the cortical areas that showed significant GM structural changes during motor training, we performed statistical tests on the results from VBM analysis. GM increase from the baseline was tested at each time-point (i.e., day 1 vs. baseline, day 5 vs. baseline, 1 week follow-up vs. baseline and 1 month follow-up vs. baseline) in a two-factor (time and participant) full-factorial test. In each statistical test, the contrast weight for the time-point was set to be −1 for baseline image, 1 for each time-point to be tested, and 0 for other time-points. The weight for each participant was also set to be 0. For statistical analysis, we excluded all voxels with a GM value <0.2 to avoid possible partial volume effects near the border between the GM and white matter. For each analysis, cluster size was corrected according to the local smoothness values, using non-stationary cluster extent correction at *P* < 0.05. We have reported the effects for clusters of voxels exceeding a voxel-level threshold of *P* < 0.001 (uncorrected) and cluster size threshold at *P* < 0.05 that were family-wise error-corrected for multiple comparisons, in the context of Gaussian random field theory.

We extracted the mean GM volume (for each time-point) using the MarsBar tool^2^ for SPM in the detected clusters on the first day that were previously subdivided using the Automated Anatomical Labeling atlas (Tzourio-Mazoyer et al., 2002). Then, the GM volume increase ratio over the baseline scan was calculated for each area.

### Regression Analysis for Behavioral and Neuroimaging Data

We performed a stepwise regression analysis using Equation 2 to investigate the relationship between GM volume changes and motor task performance:

(2) $$Y=a+b_{1}X_{1}+b_{2}X_{2}+\ldots$$

In this model, *X_i_* denotes the ratio of increase in GM volume from baseline values to the GM volume on the first day. The number of data points used for the estimation of parameters *a* and *b* (i.e., *X* and* Y*) was same as the number of participants (*N* = 14, excluding participant M, for whom the image from the first day was unavailable). In the multiple regression analysis, we included the regions where GM volume significantly increased on the first day; *Y* denotes behavioral parameters λ and *C* of the initial and endpoint errors (“*Behavioral Data Analysis*”), and λ of the peak velocity calculated by exponential fitting of the 5-day error curve. In the current study we focused on the former two parameters because we considered them to be the most meaningful for accounting for longitudinal learning effects. λ of the peak velocity was included to the analysis as a null control index. The significance level for adding/removing possible parameters was set to *P* < 0.05. We performed stepwise multiple regression analysis because the number of explanatory variables was not determined *a priori*.

## Results

### Behavioral Results

**Figure 2:**
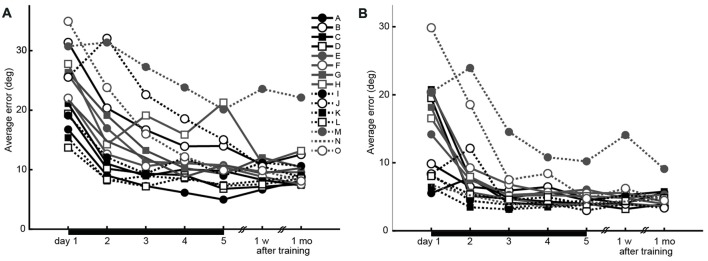
Performance changes during the experiment (15 participants). **(A)** Initial errors recorded 150 ms after movement onset, as the index for accuracy of feed-forward motor control. **(B)** Endpoint errors recorded at movement offset, as the index for the combination of feed-forward and feedback motor control accuracies. Black horizontal bars from days 1 to 5 indicate the training period.

The average time to the peak velocity from the trial onset was 211.6 ± 26.7 ms. The average peak velocity was 445.6 ± 20.3 mm/s. Both the initial and endpoint errors decreased across the 5 days of training, demonstrating that training led to significant performance improvement (two-way repeated-measures ANOVA, main effect of error type, *F*_(1,140)_ = 77.7, *P* = 4.19 × 10^−15^; main effect of day, *F*_(4,140)_ = 21.8, *P* = 5.48 × 10^−14^; interaction effect, *F*_(4,140)_ = 0.36, *P* = 0.84; Figure 2). The lack of significant interaction effect suggests that there was no difference in progression between error types. Further, there was no difference in peak velocities between the first and last days (paired *t*-test, *t*_(14)_ = −1.26, *P* = 0.22).

The estimated parameters λ and *C* for each participant, and the constant* A* estimated from the average learning curve are shown in Table 1. These values were used to analyze the relationship between motor learning and structural changes in the brain (“*Regression Analysis for Behavioral and Neuroimaging Data*”). Finally, there was no difference in the estimated parameters λ and *C* between the male and female participants (Mann-Whitney *U*-test; *P* > 0.05).

### Neuroimaging Results

**Figure 3:**
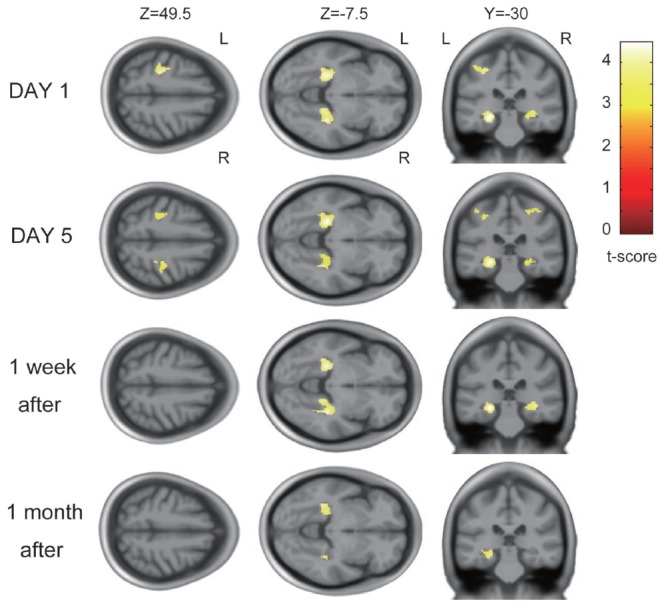
Gray matter (GM) increase across participants after visuomotor training. On the first day of training, the left primary motor cortex (M1) and primary sensory cortex (S1) hand areas and bilateral hippocampi and parahippocampi showed significant increases with learning. The same areas showed increase in GM volume on the fifth day of training. GM increases were also detected in the bilateral hippocampi and parahippocampi 1 week and 1 month after training.

On the first day of training, GM volume increases were detected in the hand area of the left M1/primary sensory cortex (peak voxel:* t* = 4.10; MNI coordinates: −28, −27, 48; spatial extent = 413 voxels) and bilateral hippocampi and parahippocampi (left peak voxel: *t* = 4.46; MNI coordinates: −26, −30, −11; spatial extent = 951 voxels; and right peak voxel: *t* = 4.21; MNI coordinates: 34, −34, −6; spatial extent = 636 voxels; *P* < 0.05, corrected for multiple comparison; Figure 3). No significant GM increases were observed in any other areas.

The areas where significant volume increases were detected on the first day changed over the course of the study. The GM increase in the left M1/S1 persisted on the fifth day of training (Figure 3) but was not detected 1 week or 1 month after training. In the bilateral hippocampi and parahippocampi, the GM increase persisted on day 5 of training and 1 week and 1 month after training. GM volume increase ratios over the baseline scan for each area are described in Supplementary Figure S1.

### Regression Results for Behavioral and Neuroimaging Data

**Figure 4:**
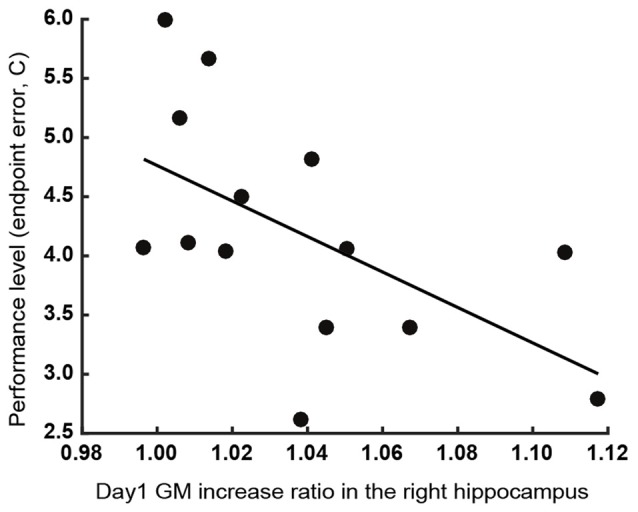
Relationship between the increase in GM volume in the right hippocampus on the first day of training and final error level (*C*) of the endpoint error. Participants whose right hippocampal GM volume exhibited a greater increase showed a lower final error level after training.

**Figure 5:**
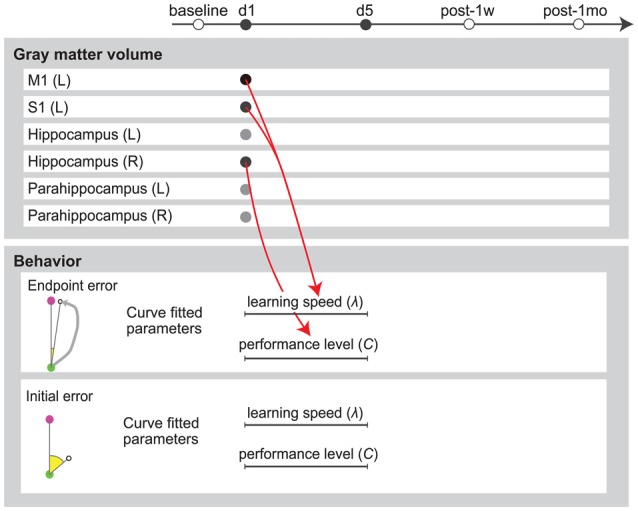
Multiple regression analysis summary. Red arrows indicate positive predictors. Circles in the upper panel indicate the ratio of increase in GM volume from baseline value. Lines in the lower panel indicate parameters calculated by exponential fitting of the 5-day error curve.

We performed stepwise multiple regression analysis to investigate the relationship between GM structural alterations and behavioral changes. For a subset of brain areas, the ratio of increase in GM volume on the first day predicted the subsequent motor learning. The ratio of increase in GM volume in the left M1 and S1 were significant predictors of learning speed (λ) of endpoint error (*P* = 0.036, *r*^2^ = 0.52, *b* = 1.95, 3.16 for the left M1 and S1). The ratio of increase in GM volume in the right hippocampus was a significant predictor of final error level (*C*) of endpoint error (*P* = 0.032, *r*^2^ = 0.33, *b* = −15.0; Figure 4). All significant combinations of the ratio of increase in GM volume and behavioral parameters are shown in Figure 5. However, none of the brain areas that showed GM increase in the first day predicted learning speed (λ) of peak velocity, suggesting that GM structural alterations exhibit functional specificity for motor learning rather than habituation for the task.

## Discussion

In this study, we used VBM to evaluate brain structural changes that occurred following novel visuomotor training. Similar to previous VBM studies (Landi et al., 2011; Sampaio-Baptista et al., 2014), we found that GM volume increased in the M1/S1, hippocampus and parahippocampus. We also detected GM structural changes induced by motor training on a short timescale (e.g., hours), which is consistent with the results of a previous report (Taubert et al., 2016). Further, we found that the volume of GM increase on the first day correlated with performance improvement after a 5-day training period.

Some recent studies have reported that structural changes, on VBM analysis, were observed only 1 h after body balancing task (Taubert et al., 2016), and on diffusion tensor imaging they were observed 2 h after a spatial learning and memory task in both humans and rats (Sagi et al., 2012; Hofstetter et al., 2013). Compared with these investigations, the current study is novel because our results show that the degree of rapid structural plasticity predicts subsequent motor learning. Importantly, the participants whose GM structure did not change exhibited less motor performance improvement, suggesting a relationship between GM structural changes and future motor learning.

### Localized GM Changes Associated With Visuomotor Training

Consistent with previous reports (Landi et al., 2011; Sampaio-Baptista et al., 2014), we detected GM volume increases in the M1 and S1, which are involved in motor execution (Nudo et al., 1996; Scott et al., 2001; Matyas et al., 2010; Pruszynski et al., 2011), motor memory (Robertson, 2012) and skill acquisition (Karni et al., 1998; Lohse et al., 2014). The GM increases in the M1 and S1 were correlated with learning speed (λ) of endpoint error, which seem reasonable since M1 and S1 are assumed to be involved in feedback control, as traditionally suggested (Lacquaniti and Maioli, 1989). Thus, brain changes in this area may contribute to visuomotor learning. In addition, we also detected GM increase in the hippocampus and parahippocampus, which have not been reported earlier in human motor learning studies, such as studies involving visuomotor rotation tasks (Landi et al., 2011). Unlike a rotation task, a mirror reversal task requires more explicit (i.e., declarative) strategy (Telgen et al., 2014) to aim at the memorized mirror-reversed position of the visual target. Such type of learning is considered to be a model of motor skill learning (Shmuelof et al., 2012, 2014), which is distinct from error-based forward model adaptation (Diedrichsen et al., 2005). According to the previous studies reporting that hippocampus and parahippocampus are involved in declarative and spatial memory (Nadel and Moscovitch, 1997; Leutgeb et al., 2005), these areas are considerably activated when the participants imagined making reaching movements toward the mirror-reversed direction of the presented target or pointed to the memorized target located at the mirror-reversed target position. Consistent with this hypothesis, animal studies have demonstrated that hippocampal inactivation impaired motor skill learning (Wächter et al., 2010; Yang et al., 2014).

We acknowledge that it is not possible to conclude that the mirror-reversal task was necessary for these structural changes because we did not instruct the participants to perform repetitive normal reaching without any virtual transformation. However, our findings indicate that the structural changes detected by MRI could reflect the functional contribution of the M1/S1 cortices and hippocampal areas in a repetitive arm-reaching task. While the results would have been more convincing if we had included a control group (i.e., motor activity without learning) and distinguished between regional GM volume changes driven by mere motor activity vs. GM changes due to visuomotor learning, we can assume that at least a part of the GM changes was driven by visuomotor learning because there was a significant correlation between the GM changes and visuomotor task performance. The result demonstrating no relationship between the changes in peak velocities (i.e., a null control index that is independent of learning) and the GM increases also supports this assumption.

The present study shows the impact of the GM change on the first day on the subsequent behavioral performance during motor learning. Further comprehensive analysis is needed to reveal the complete timescale of the relationship between brain structure and behavior, while considering their interaction, such as performance improvement due to volume increase and volume changes due to experience.

### Neurobiological Candidates for Rapid GM Increase

The GM increase detected using VBM could be due to different signal intensities as a result of changes in the brain tissue composition. Such alterations can occur within a day, whereas it is unlikely that morphological changes occur on such a short timescale. Although it is difficult to clarify the underlying biological mechanisms resulting in increased GM signal on MRI, recent animal studies have provided new molecular and cellular evidence of experience-dependent GM structural changes (Kleim et al., 2002; Dong and Greenough, 2004; Kolb et al., 2008; Zatorre et al., 2012). The cellular-level candidates include neurogenesis, axon sprouting, dendritic branching, synaptogenesis, glial swelling, glial increase, vascular volume change and angiogenesis (Zatorre et al., 2012). Among these, glial swelling and vascular volume changes are thought to occur within minutes to hours (Macvicar et al., 2002; Takano et al., 2006; Theodosis et al., 2008); thus, the signal changes detected in the current study may be attributed to these cellular mechanisms.

### Candidate Mechanism Underlying the Relationship Between Performance Improvement and Initial GM Increase

The most insightful finding of the current study is that performance improvement (i.e., functional change) can be predicted by the initial increase in GM volume (i.e., structural change). Although the current study design was insufficient to provide evidence for a causal relationship between the two, if we assume that the increased GM signal observed was due to structural changes in glia and blood vessels, this finding could be the result of structural plasticity, inducing subsequent neural plasticity or functional changes. In support of this hypothesis, a recent study has revealed that a structural change in the astrocytic domains, in response to increased neural activity, promoted excitatory synapse stability (Bernardinelli et al., 2014). Astrocytic plasticity has also been proposed to play critical roles during dendritic spine maturation (Nishida and Okabe, 2007) and synapse elimination (Chung et al., 2013). Moreover, astrocytes mediate vasodilation, and help to increase the supply of neuronal metabolites (Takano et al., 2006), such as lactate, an organic chemical that is necessary for memory and learning (Newman et al., 2011).

### Limitations

This study has several limitations. First, VBM is less accurate in normalization and segmentation processes than surface-based algorithms (Lerch et al., 2017). In addition, due to the difference in the deformation procedure, the smoothness of the VBM signals is inferior to that of surface-based algorithms, and thus, statistical sensitivity may also decrease (Lerch et al., 2017). Second, the large number of regression models tested and predictors included in the model may inflate the risk of type I error. Therefore, although we still cannot completely eliminate the risk of type I error, in the current study we reduced the number of models and predictors to five behavioral parameters that would be most meaningful for describing performance changes, and six brain regions that showed significant increase in GM volumes from baseline to the first day of training. Third, we should also be cautions when interpreting our results, because the longitudinal analysis tools in VBM8 can be biased toward a particular MRI time-point. However, in this study we focused on short-term structural changes, where large-scale changes which modify a deformation field are not known to occur. In such a case, separate preprocessing for each scanning time-point, which generates a separate deformation field for each normalization process, may not be appropriate because it increases the risk of artifact formation. Therefore, we consider it better to use longitudinal preprocessing with a unique deformation field that is common between time-points, in order to reduce the normalization error between time-points. In addition, the age range (20–23 years old) of the participants enrolled in this study is narrow. Finally, we should also be cautions in interpreting results from this and other VBM studies because GM volume is affected by hydration status and fasting.

## Conclusion

In the current study, we found that GM structural changes in areas involved in visuomotor learning were detected even after the first training session in a mirror-reversal experimental setup. Moreover, the degree of increase in the GM volume noted in the first MR scan was associated with the performance assessed after 5 days of training. Our findings demonstrate that structural changes are not simply a footprint of previous motor learning; rather, they are related with future motor learning. Individual differences in GM structural changes and behavioral improvements are important questions for future studies. The predictability of motor learning consequences, based on initial structural changes, may be applied to develop novel training regimes in sports and rehabilitation, with the potential to considerably increase the benefit of practice.

## Author Contributions

MK, SK and JU designed the research. MK, SK, TO, FY, HE, ML and JU contributed unpublished reagents/analytic tools and discussed the results and finalized the manuscript. MK performed research. MK, SK and FY analyzed the data. SK, MK, FY and JU wrote the article.

## Conflict of Interest Statement

The authors declare that the research was conducted in the absence of any commercial or financial relationships that could be construed as a potential conflict of interest.

## Footnotes

^1^http://dbm.neuro.uni-jena.de/vbm8/

^2^http://marsbar.sourceforge.net/

## Supplementary Material

FIGURE S1 The increase ratio of gray matter (GM) volume over the baseline scan in each subdivided area using the Automated Anatomical Labeling atlas; **(A)** left M1, **(B)** left primary sensory cortex (S1), **(C)** left hippocampus **(D)** right hippocampus, **(E)** left parahippocampus and **(F)** right parahippocampus.
