# Supplementary figures and images for: Structural Gray Matter Changes in the Hippocampus and the Primary Motor Cortex on An-Hour-to-One- Day Scale Can Predict Arm-Reaching Performance Improvement
Source: Front Hum Neurosci. 2018 Jun 8;12:209. doi: 10.3389/fnhum.2018.00209 (PMC6024594; doi:10.3389/fnhum.2018.00209)

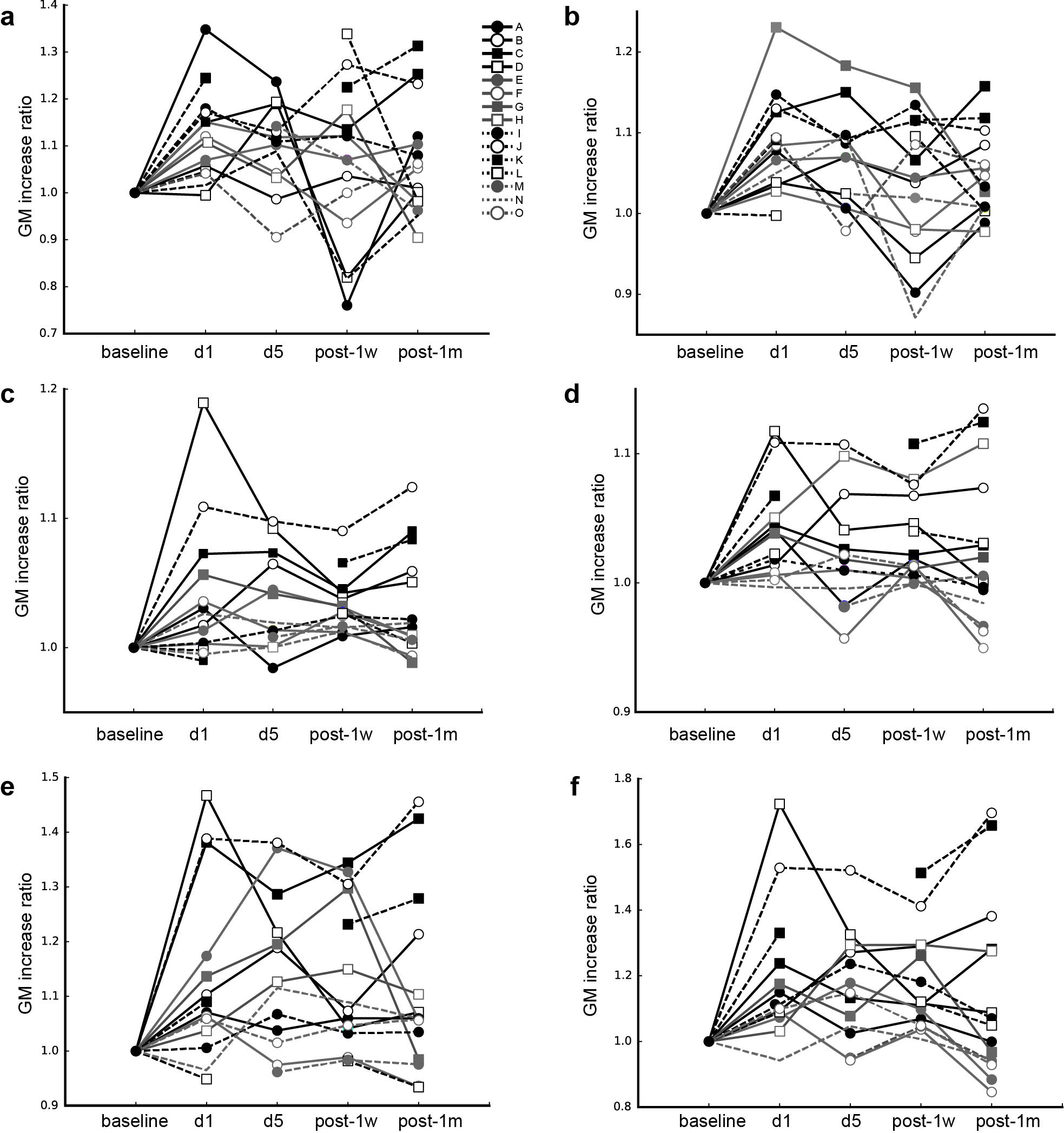

Supplement: FIGURE S1 — The increase ratio of gray matter (GM) volume over the baseline scan in each subdivided area using the Automated Anatomical Labeling atlas; (A) left M1, (B) left primary sensory cortex (S1), (C) left hippocampus (D) right hippocampus, (E) left parahippocampus and (F) right parahippocampus. [file Image_1.TIF]
